# Establishing an independent HTA agency in Ukraine: a conceptual framework for governance, operations, and long-term sustainability

**DOI:** 10.1017/S0266462325103255

**Published:** 2025-12-11

**Authors:** Rabia Sucu, Daniel Erku, Olena Filiniuk, Rebecca Kohler

**Affiliations:** 1 Health Economics and Financing, Global Health Systems Innovation, Management Sciences for Health, USA; 2 Economic, Policy and Innovation Centre (EPIC) for Health Systems, Ethiopia; 3 Centre for Applied Health Economics, School of Medicine, Griffith University, Australia; 4 SAFEMed, Management Sciences for Health, Ukraine

**Keywords:** health technology assessment, Ukraine, health policy, governance, priority setting in health

## Abstract

**Objectives:**

Health technology assessment (HTA) has become an integral part of Ukraine’s health system since its formal introduction into national legislation in 2017. By 2020, HTA was mandated for evaluating publicly funded medicines, laying the groundwork for more evidence-based healthcare decisions. Although the creation of an independent HTA agency was initially planned for 2022, implementation was delayed due to the COVID-19 pandemic and Russia’s ongoing invasion. The relevant Cabinet Resolution calls for the establishment of an autonomous agency by January 2026. This commentary outlines a strategic, evidence-informed framework to guide the agency’s formation.

**Methods:**

Drawing on the 2018 State Strategy for Access to Medicines, the 2022 Law on Medicinal Products, and international best practices, we proposed to the Government of Ukraine a two-tier structure encompassing *core business* functions (HTA and appraisal, guideline development, pricing, and listing) and *support business* functions (data and analytics, finance and strategy, IT, human resources, legal, and communications). Each department is tasked with clear mandates and supported by performance indicators to promote transparency, accountability, and operational efficiency.

**Results:**

A phased roadmap for 2025–2027 details the legal, institutional, and financial steps required for successful implementation. Key opportunities – including international partnerships and system-wide reform – are weighed alongside risks such as funding uncertainty, workforce limitations, and geopolitical instability.

**Conclusion:**

By embedding HTA into national policy processes and ensuring institutional independence, Ukraine can enhance the value of healthcare investments and build long-term resilience into its health system.

## Introduction

Health technology assessment (HTA) was formally integrated into Ukraine’s national healthcare legislation in 2017 (1), laying the foundation for a more evidence-informed approach to health policy. The institutionalization process began in January 2019 with the creation of an HTA department within the State Expert Center (SEC) under the Ministry of Health (MoH). In 2020, HTA became a mandatory requirement for evaluating medicinal products financed through public funds. A key strategic objective of the Ukrainian government is to establish an independent HTA agency as part of broader institutional reforms aimed at ensuring access to high-quality, effective, and safe medicines. This vision aligns with the Cabinet of Ministers’ Resolution published in December 2020 (2), which mandates the MoH to take steps toward establishing a state unitary commercial enterprise dedicated to conducting HTA by January 2026.

The independent HTA agency is expected to be established through the planned restructuring of the SEC. This restructuring offers a timely and strategic opportunity for the existing HTA unit to evolve into a fully autonomous agency with a dedicated mandate. Supporting this transition, the 2022 Law “On Medicinal Products” (3) provides for the establishment of a State Control Authority (SCA) – the national regulatory body to become operational in January 2027 – tasked with overseeing medicinal product policy, including quality, safety, and efficacy. The future HTA agency is envisioned to operate alongside this structure with clear mandates, operational independence, and a strong legal foundation.

In establishing the agency, Ukraine draws on lessons from international HTA systems, which typically follow a two-phase process: evidence assessment and appraisal. While structures differ, most systems combine clinical and economic evaluations with broader considerations – such as patient impact and budget implications – to guide coverage decisions. Several agencies, such as in the United Kingdom, Canada, and Germany, offer examples of institutional independence, transparent processes, and effective stakeholder engagement. Topic selection approaches range from industry-driven submissions to government-led horizon scanning, with hybrid models (e.g., the Netherlands, Canada) balancing near-term needs with long-term priorities.

Timeliness, flexibility, and inclusivity are core features of mature HTA systems. The European Union (EU) Transparency Directive (4), for instance, sets a 180-day window for pricing and reimbursement decisions. Countries such as the Netherlands, Germany, and the United Kingdom have achieved rapid yet robust HTA timelines, often supported by mechanisms like managed entry agreements (MEAs) or conditional reimbursement pathways that address uncertainty while ensuring early access. Effective stakeholder engagement – including from industry, clinicians, and patients – further enhances legitimacy and utility.

This Concept Note draws on Ukraine’s legal and institutional context, global best practices, and the Integrated Business Plan developed under the United States Agency for International Development (USAID)-funded “Safe, Affordable and Effective Medicines for Ukrainians (SAFEMed) project,” managed by Management Sciences for Health (MSH). It proposes a conceptual framework for establishing a sustainable HTA agency – covering governance, structure, functional domains, performance metrics, funding models, and a phased implementation roadmap. Ultimately, establishing a fully independent agency based on the existing HTA function can enhance Ukraine’s policy processes, promote independence and transparency, and align with international standards. This approach can support the delivery of equitable, evidence-based health care while strengthening system resilience amid ongoing fiscal and geopolitical challenges.

## Legal framework for establishing an independent HTA agency

The legal foundation for HTA in Ukraine was established through the *Fundamentals of Ukrainian Legislation on Healthcare* (5), originally enacted in 1992 and subsequently revised multiple times through 2025. This legislation formally defines HTA and mandates its application in managed entry agreements (MEAs) as well as in the development of sectoral standards across the healthcare system.

This framework was strengthened by a Cabinet Resolution in 2018, which approved the State Strategy for Ensuring Access to Medicines through 2025 (6). The strategy calls for the establishment of a comprehensive HTA-based system for medicine selection and the regular, transparent updating of the National Essential Medicines List (EML) based on HTA principles.

The operational procedure for conducting state-level HTA was codified in the 2020 Cabinet Resolution (2), which outlines the responsibilities of executive agencies, defines submission and review requirements for HTA dossiers, and sets criteria for decision making. This resolution also tasks the MoH with establishing a dedicated state agency to conduct HTAs by 1 January 2026.

At the request of the Ukrainian government, the SAFEMed technical assistance project drafted a comprehensive legal package to support the establishment of an independent HTA agency in Ukraine and submitted it for further government consideration. This package includes a Charter as well as detailed regulations and annexes outlining an open and merit-based process for selecting the Agency’s Director – complete with confidentiality provisions and conflict-of-interest safeguards. It also contains templates for public announcements and application materials for prospective leadership candidates.

## Purpose and scope of the HTA agency

The primary mandate of the HTA agency is to ensure that limited public resources are allocated to health interventions offering the greatest value. This involves the systematic evaluation of medicines, medical devices, diagnostics, and public health programs based on clinical effectiveness, safety, and cost-effectiveness. The Agency’s assessments will guide the MoH in decisions about which technologies should be included in or excluded from the National Essential Medicines List (Single Positive List), as well as identifying candidates for disinvestment – technologies that are outdated or no longer cost-effective.

In addition to its assessment and appraisal functions, the proposed Agency will play a central role in developing national clinical guidelines and sectoral standards. These include protocols for acute, chronic, and rehabilitative care, formularies, and technical specifications for equipment, all designed to harmonize clinical practice across the country and promote evidence-based care delivery. The Agency also will advise on mechanisms to manage uncertainty in emerging technologies, such as early-access provisions, managed entry agreements, and risk-sharing arrangements – helping the health system to balance innovation with sustainability.

The current department’s responsibilities are limited primarily to medicines and play only a minor role in guideline development. With the establishment of a new agency, these responsibilities will expand to include nonmedicine technologies, horizon scanning, and a strengthened role in managed entry agreements, while also ensuring greater independence and transparency. Administratively, the agency will be responsible for maintaining the Single Positive List and for developing pricing proposals that reflect assessed value while safeguarding equitable access to essential health products in publicly funded settings.

## Stakeholders and end users

Agency’s work serves a broad spectrum of stakeholders, besides MOH. The Ukrainian MOH and other government entities and public health institutions use HTA outputs to inform national policy and funding allocations. Healthcare providers – including hospitals, clinics, and individual practitioners – rely on its clinical guidelines and appraisals to guide treatment decisions. Payers and insurers reference HTA findings to shape benefit design and coverage policies that prioritize value for money. The pharmaceutical and medical device industries use HTA processes to inform their market-entry strategies and pricing expectations. International organizations engage the Agency in technical collaborations, while universities and research institutions contribute to and draw from its growing body of analytical work. Importantly, patients, civil society groups, and the public depend on the Agency’s transparent methodologies and publicly accessible outputs to better understand which health interventions are supported by evidence and why. The existing HTA function, in its more than 5 years of history in Ukraine, has successfully engaged a wide range of stakeholders to raise awareness and encourage participation. With the establishment of an independent agency and an expanded mandate, HTA will serve as an even more trusted resource across all stakeholder groups, with a stronger impact on policy, practice, and public confidence.

## Organizational structure and functional areas

**Figure 1.**
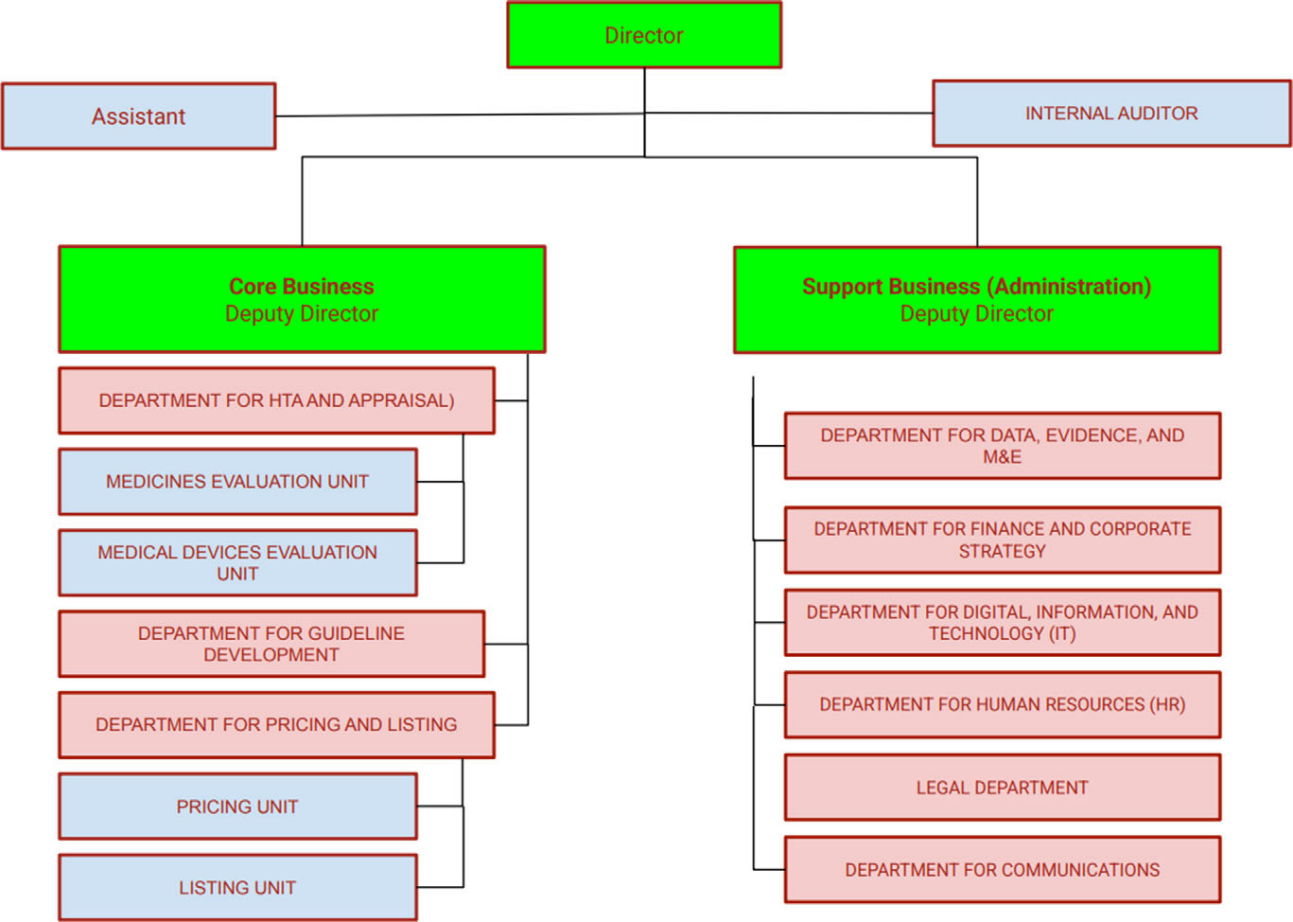
Organizational structure of the proposed HTA agency.

The proposed agency structure is organized around two functional pillars – Core Business and Support Business – to fulfill both its technical and operational mandates effectively. Leadership and coordination are maintained through a centralized management structure that ensures clear lines of accountability across all departments. The focus is on the essential functions for the launch of the agency, with an assumption that functions may be expanded or revised over time based on needs and conditions. Figure 1 provides an overview of the Agency’s organizational structure, and Table 1 summarizes the core and support functions along with their associated key performance indicators.

**Table 1.** Departments of the HTA agency: Core and support functions with key performance indicators

Department/unit	Main functions	Key performance indicators
Core business functions		
Department for Health Technology Assessment (HTA) and Appraisal	Evaluates submissions of new health technologies; manages inclusion/exclusion of medicines on the Single Positive List; identifies technologies for disinvestment; supports managed access agreements; evaluates updates to public health programs and interventions to decide on uptake, disinvestment, or revision.	Average time from receipt of submission to publication of assessment conclusion Stakeholder satisfaction (providers, patients, industry) via annual surveys % of rapid assessments completed within 30-day mandate Economic benefits realized (cost savings or cost avoidance) % of HTA recommendations uptake by the Ministry of Health Number of technologies recommended for disinvestment due to lack of efficacy or cost-effectiveness and impact
*Medicines Evaluation Unit*	Detailed assessment and appraisal of medicines – efficacy, safety, cost-effectiveness, and other aspects – to support decisions on their use in the healthcare system.	
*Medical Devices Evaluation Unit*	Comprehensive evaluations of medical devices – comparative efficacy, safety, economic value, and other aspects – to support decisions on their adoption and use within the healthcare framework.	
Department for Guideline Development	Develops clinical guidelines, medical care standards, rehabilitation care protocols, equipment and formulary lists, and other medical–technical documents based on systematic reviews and expert consensus; creates educational materials and workshops to inform providers about new guidelines and updates.	% of standards and protocols aligned with national/international best practices Frequency and timeliness of guideline updates Average time to develop/revise/approve standards % of documents updated on schedule Number of expert consultations/feedback sessions % of providers reporting full implementation Impact on patient outcomes (e.g., treatment errors, rehabilitation success) # of professionals trained on new guidelines/protocols % of facilities adhering to formularies/equipment lists # of discrepancies found in audits Cost-effectiveness of standard implementation # of collaborations with WHO or other bodies Effectiveness of feedback integration into revisions
Department for Pricing and Listing	Manages and regulates health-technology pricing; maintains the Single Positive List; ensures accurate, value-reflective pricing that maintains affordability for state-funded institutions.	Average time to set price and list new medicines Effectiveness of ERP/IRP in balancing international competitiveness and economic feasibility Annual budget impact of newly listed medicines % of medicines on Positive List based on HTA and WHO list – Frequency of Positive List updates Affordability index relative to household income # of stakeholder consultations on pricing/listing
*Pricing Unit*	Implements external reference pricing (ERP) and internal reference pricing (IRP); recommends value-based prices to the Ministry of Health; updates price lists per MoH decisions.	
*Listing Unit*	Maintains and regularly updates the Single Positive List based on Ministry of Health decisions.	
Support business functions		
Department for Data, Evidence and Monitoring and Evaluation (M&E)	Integrates latest scientific theories into HTA processes; conducts research on price trends, utilization, expenditure tracking; horizon scanning for emerging technologies; develops performance measurement tools to monitor program effectiveness and efficiency.	Success rates of agency programs versus planned objectives Impact of research on HTA processes (data quality, utilization, methodology adoption) # of citations of published research Adoption rate of new methodologies Feedback from internal departments Effectiveness of collaborations (joint publications, shared projects, partner feedback)
Department for Finance and Corporate Strategy	Conducts data collection on price trends, utilization, and expenditure; horizon scanning for emerging health innovations; integrates findings into corporate strategy and budgeting.	Budget adherence: forecast versus actual expenditures Financial efficiency: cost-saving initiative impact
Department for Digital, Information and Technology (IT)	Designs and maintains user-friendly digital platforms; manages IT infrastructure and operations; ensures security, data integrity, and system reliability.	System reliability (uptime and availability) User satisfaction with digital tools (internal and external)
Department for Human Resources (HR)	Talent acquisition and recruitment aligned with organizational goals; employee onboarding and training.	Average time to fill open positions – Employee turnover rate Performance and retention rate of new hires Employee participation in development programs Employee engagement levels via surveys
Legal Department	Protects legal compliance and manages risk; handles contract management, corporate governance, legislation, and intellectual property.	# of legal documents processed per period Average response time to stakeholder inquiries Contract cycle time % of legal cases won or settled favorably # of legal issues prevented through advice Stakeholder satisfaction with legal services
Department for Communications	Manages public relations to enhance the agency’s image; leads consumer engagement to incorporate patient perspectives; develops strategic partnerships and international collaboration in HTA.	Responsiveness to healthcare policy changes Reach and impact of communication campaigns Engagement metrics (press mentions, social media, viewership) Volume and nature of consumer feedback Effectiveness of consumer engagement on HTA processes Strength and productivity of strategic partnerships Outcomes of international collaborations (joint projects, publications, events)
Internal Audit Unit	Conducts risk assessment and internal controls evaluation; performs financial, compliance, operational, and performance audits; fraud detection and prevention; reporting and recommendations.	% of planned audits completed on schedule % of audit findings addressed within agreed timelines % of high-risk audits completed timely # of new risks identified % of follow-up audits completed % of costs recovered through audit activities

The Core Business pillar comprises three departments. The HTA and Appraisal Department is responsible for conducting full and rapid assessments of health technologies, synthesizing clinical and economic evidence to inform decision making. The Guideline Development Department formulates and updates clinical standards, care protocols, and essential medicines formularies. The Pricing and Listing Department manages the Single Positive List and provides recommendations on value-based pricing for publicly funded technologies.

The Support Business pillar includes six departments with associated units that provide essential infrastructure and services. The Data, Evidence and Analytics Department supports evidence generation by collecting and analyzing data on technology use, costs, and outcomes. The Finance and Corporate Strategy Department oversees budgeting, funding strategies, and long-term planning. The Digital, Information and Technology Department maintains the Agency’s IT systems and digital platforms. The Human Resources Department manages recruitment, capacity building, and performance evaluation. The Legal Department ensures regulatory compliance, manages contracts, and mitigates legal risks. The Communications Department handles stakeholder engagement, public outreach, and dissemination of HTA findings. This organizational structure supports timely, transparent, and high-quality outputs and provides a foundation for the Agency to evolve into a credible, independent institution capable of supporting evidence-informed policy decisions in Ukraine’s health system.

## HTA agency roadmap

**Figure 2.**
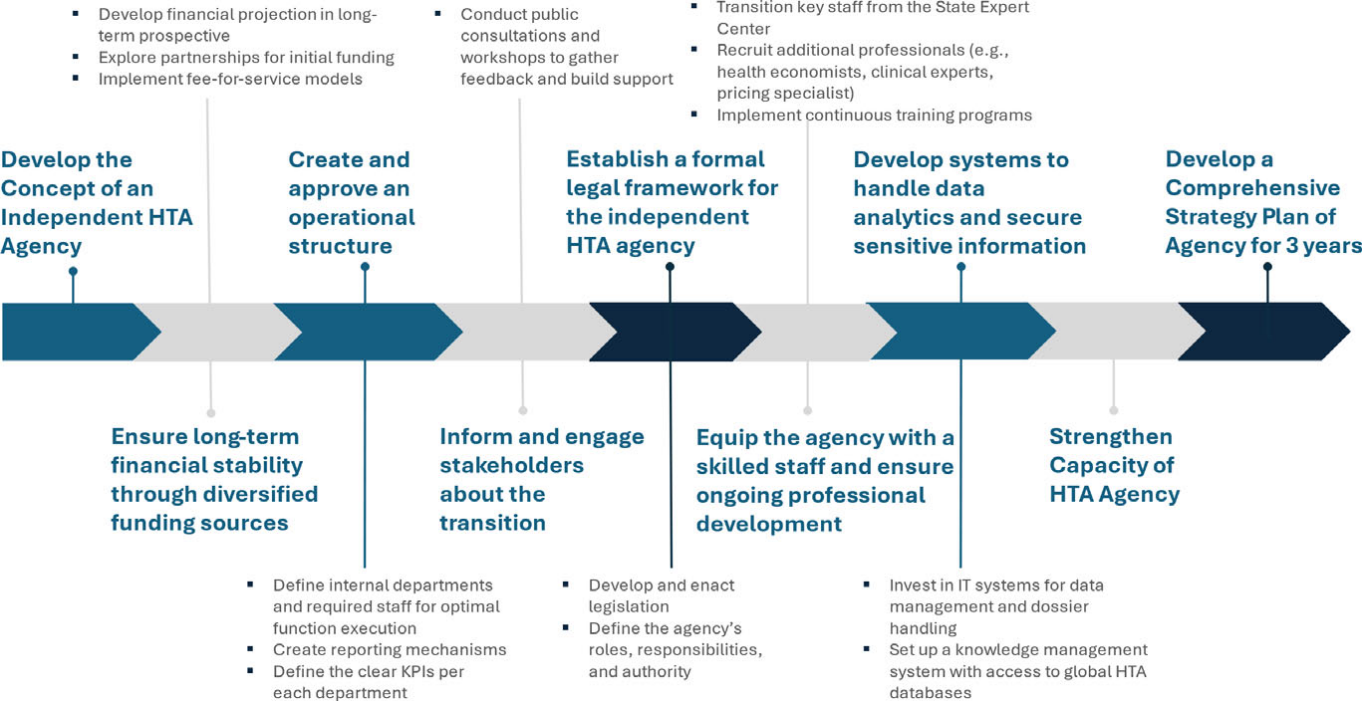
HTA agency roadmap.

Implementation of HTA function in Ukraine followed a detailed roadmap for HTA institutionalization that was created in 2019. While this roadmap was very helpful to raise awareness, develop capacity, set the legal framework for including HTA in legislations and integrate it into healthcare ecosystem in Ukraine, creation of the independent HTA agency was believed to require a unique roadmap for its own. Transitioning from the current HTA department within the State Expert Center to a fully independent agency required a phased approach that preserves ongoing assessments while building the new institution’s legal, financial, and operational foundations. Figure 2 outlines a proposed roadmap structured around three strategic phases: Preparation, Launch, and Stabilization and Scale-Up.

In the *Preparation phase* (through the formal date of launch), the priority is to put in place the legal and governance framework. Under the SAFEMed project’s draft Charter and regulations, the State Expert Center will be formally renamed and its roles transferred to the new Agency. At the same time, a blended funding model – combining state budget allocations, submission fees, and donor grants – must be agreed and initial budget lines secured. Open, merit-based recruitment for the Director, Deputy Directors, and other key leadership positions will ensure that the Agency inherits experienced staff while embedding conflict-of-interest safeguards into its selection processes.

Once those elements are in place, the *Launch phase* will bring core functions into operation. Three Core Business departments – HTA and Appraisal, Guideline Development, and Pricing and Listing – will begin work alongside the five Support Business departments (Data and Analytics; Finance and Strategy; IT; Human Resources; Legal; and Communications). Early activities will include rapid-assessment projects, the publication of a prioritized topic list, finalize and publicize its first set of clinical guidelines and pricing rules for the Single Positive List. Establishing routine workflows for dossier submission, evidence appraisal and committee deliberations will be critical to demonstrating operational capability.

Beginning in 2027 and beyond, the *Stabilization and Scale-Up* phase will focus on expanding capacity and embedding continuous improvement. Full-assessment teams and horizon-scanning units will be strengthened, and performance-based budgeting will be refined to include annual fee schedules tied to submission volumes. The Agency will deepen its international collaborations – participating in EU joint assessments and exchanging best practices through INAHTA, HTAi, and ISPOR networks – to accelerate learning and credibility. Annual governance, staffing, and funding reviews will allow the Agency to adjust to changing needs and to shore up resilience against geopolitical and fiscal shocks.

Throughout each phase, a core set of Key Performance Indicators among those listed earlier – such as time from dossier receipt to recommendation, stakeholder satisfaction levels, and adherence to budget forecasts – will be monitored closely. Regular progress reviews, conducted in partnership with the stakeholders, will ensure that any emerging challenges are addressed promptly and that ongoing HTA activities proceed without interruption. By maintaining decision-making continuity, securing sustainable financing and strengthening institutional capacity, this roadmap will guide the creation of a transparent, autonomous and resilient HTA agency equipped to meet Ukraine’s health-technology needs now and in the future.

## Funding models for the independent HTA agency

Establishing a financially sustainable HTA agency in Ukraine will require a carefully balanced funding strategy. International experience shows that HTA bodies vary widely in how they are funded and resourced, depending on national contexts, institutional maturity, and assessment volume. Where internal capacity is still developing but demand for HTA is high, agencies often begin with a hybrid model – relying on core public funding while gradually building technical capability and exploring supplementary revenue streams.

A 2018 European Commission review of 56 HTA bodies across 27 EU Member States and Norway (7) found that nearly all rely primarily on government budgets, though some also generate revenue through service fees. Examples include Poland’s AOTMiT (submission fees), NICE in the United Kingdom (scientific advice fees), and Germany’s G-BA (early-dialogue fees), with others such as Latvia and Scotland reporting occasional consultancy income. Despite these models, almost half of agencies surveyed did not disclose budget information, underscoring the need for greater transparency in HTA funding and capacity. As shown in Table 2, staff sizes, turnaround times, and per-assessment costs vary widely between countries, even when adjusted for health system scale (8).

**Table 2.** Required HTA capacity and costs in various countries

Country	Entity	Responsibilities	Number of staff	Time required to produce an HTA	Cost per HTA (2011 US$)	Operating budget (2011 US$)
Australia	Pharmaceutical Benefits Advisory Committee (PBAC)	Assess pharmaceutical dossiers	18 members; > 40 support staff	8–9 weeks	~ 60,000	15 million (0.01% of statutory health insurance budget)
	Medical Services Advisory Committee (MSAC)	Evaluate medical services	4 executives; 20 staff	13 months (12 evaluations/year)	~ 250,000	Not available
	Prostheses List Advisory Committee	Update the prostheses list	16 staff	Semi-annual updates	Not available	Not available
Germany	Institut für Qualität und Wirtschaftlichkeit (IQWiG)	Produce comprehensive and rapid HTA reports and dossier assessments	122 (in 2011)	Full: 18 months; Rapid: 4–6 months; Dossier: 3 months	65,000–650,000	19 million (0.01% of statutory health insurance budget)
Poland	Agency for Health Technology Assessment (AOTMiT)	Prepare HTA-based financing recommendations for public funds; advise local governments	~ 55	2–3 months	28,000–43,000	3.8 million (0.018% of national Health Fund budget)
United Kingdom	National Institute for Health and Care Excellence (NICE)	Evaluate and issue guidelines on technologies, pharmaceuticals and medical practice	> 650	7–14 months	320,000–400,000 (guideline); 90,000–230,000 (systematic review)	~ 90 million (0.06% of National Health Service annual budget)

To inform financial planning for Ukraine’s Agency, a five-year funding projection model was developed. Given current economic and institutional conditions, full cost-recovery is unlikely in the near term. Initial operations will depend largely on government allocations, with donor assistance, modest submission fees or training fees helping to offset startup costs. Over time, as the Agency matures and its processes become more efficient, the goal is to reduce reliance on state funding by increasing its share of internally generated revenue. Table 3 summarizes projected funding allocation across the Agency’s first five years, accounting for operational scaling, staffing needs, and anticipated income sources. This blended approach – rooted in public investment but designed for long-term financial independence – aims to ensure the Agency’s stability, credibility, and effectiveness within Ukraine’s broader health system reforms.

**Table 3.** Projected five-year funding allocation

Funding source/item	Y1	Y2	Y3	Y4	Y5
Total financing	44,543,641	51,060,759	58,637,036	66,687,661	77,907,925
Own budget (UAH)	9,545,845	16,642,630	27,792,260	40,933,005	52,522,803
– Own budget (%)	21.4%	32.6%	47.4%	61.4%	67.4%
State budget (UAH)	34,997,796	34,418,129	30,844,775	25,754,656	25,385,122
– State budget (%)	78.6%	67.4%	52.6%	38.6%	32.6%
Fundraising: IT system development (UAH)	2,500,000	N/A	N/A	N/A	N/A

## Potential opportunities and threats in establishing an independent HTA agency

The transition from a department within the State Expert Center to a fully independent HTA agency presents both significant opportunities and critical challenges that must be addressed to ensure long-term sustainability and impact. On the opportunity side, Ukraine’s ongoing financial pressures provide a compelling rationale for institutionalizing HTA as a core mechanism for allocating scarce healthcare resources. HTA can support national goals such as achieving Universal Health Coverage by ensuring that public funds are directed toward high-value interventions with demonstrable health benefits. In parallel, the legislative restructuring initiated by the 2022 Law on Medicinal Products establishes a new State Control Authority by January 2027 and provides a legal and institutional pathway for the HTA department to transition into an autonomous agency with a dedicated mandate. Internationally, Ukraine’s existing and past ties with organizations such as INAHTA, HTAi, ISPOR, and EUnetHTA offer rich opportunities for capacity building, methodological alignment, and collaborative learning. These networks also help position Ukraine as an emerging contributor to the global HTA community.

However, the path forward is not without risk. Unrealistic expectations about the new agency’s capacity could lead to overcommitment, underperformance, and a loss of credibility. Ensuring a qualified leadership team and investing in workforce development will be essential to prevent inefficiencies and staff turnover. A lack of structured HTA training programs within the country remains a limiting factor in scaling technical expertise. Furthermore, the agency may face functional overlaps with other institutions – particularly the new State Control Authority – unless roles are clearly defined in law and practice. Without strong legal safeguards, the agency’s operational independence may be vulnerable to political or institutional interference. Another threat lies in the weak link between HTA-generated evidence and actual policy implementation. Resistance from providers, policymakers, or industry actors can dilute the agency’s influence, especially if HTA outputs are not systematically used in coverage and pricing decisions. Financial constraints are also a pressing concern; without stable and sufficient funding – and a leadership team capable of managing diverse revenue streams – the agency may struggle to maintain assessment quality and operational continuity. Finally, geopolitical instability, including the ongoing war and potential regional disruptions, could divert resources and attention away from HTA, delaying its integration into health policy processes and undermining institutional momentum. Anticipating and planning for these risks are critical. By leveraging its strong legal foundation, international partnerships, and commitment to transparency, Ukraine’s emerging HTA agency has the potential to become a key pillar of evidence-based policymaking. However, this potential will only be realized through strategic investment in capacity, governance, and sustained stakeholder engagement.

## Conclusion and policy implications

This article outlines a proposal strategy that the Ukrainian government can use as a blueprint for establishing an independent HTA agency. The establishment of the agency presents a strategic opportunity for Ukraine to institutionalize evidence-based decision making and strengthen the efficiency and equity of its health system. Building on the progress made through the HTA unit within the State Expert Center and supported by a robust legal and policy framework, the transition to a fully autonomous agency can elevate HTA from a technical function to a core pillar of health governance. This shift will enable better prioritization of health spending, support the implementation of value-based pricing, and improve access to high-impact interventions.

To ensure the agency’s long-term effectiveness, three policy priorities stand out. (1) Independence and clarity of roles through strong legal and regulatory frameworks, especially in relation to the new State Control Authority. (2) A phased transition roadmap that maintains continuity while building new capacity. (3) Sustainable financing via a blended model of state funding, submission fees, and donor support.

At the same time, investing in workforce skills, strengthening national data systems, and engaging clinicians, patients, academics, and global HTA networks will reinforce credibility and impact. With political will and targeted investment, Ukraine’s HTA agency can help build a more resilient, transparent, and accountable health system.

## Data Availability

The views expressed in this article are those of the authors and do not necessarily reflect the views of USAID or the United States Government.
